# Incidence trends of prostate cancer in East Anglia, before and during the era of PSA diagnostic testing

**DOI:** 10.1038/sj.bjc.6603247

**Published:** 2006-07-11

**Authors:** N Pashayan, J Powles, C Brown, S W Duffy

**Affiliations:** 1 Department of Public Health and Primary Care, Institute of Public Health, Cambridge, CB2 2SR, UK; 2 Eastern Cancer Registration and Information Centre, Addenbrookes, Cambridge, CB2 2QQ, UK; 3 Cancer Research UK, Centre for Epidemiology, Mathematics & Statistics, Wolfson Institute of Preventive Medicine, London EC1M 6BQ, UK

**Keywords:** prostate cancer, incidence trends, age-period-cohort analysis, extrapolation

## Abstract

We investigated prostate cancer incidence in East Anglia from 1971 to 2000. Using age-period-cohort modelling, the number of cases expected in 1991–2000, based on pre-PSA trends, 1971–1990, was compared with that observed. Based on pre-1991 trends, 9203 new cases were expected in 1991–2000, but 9788 cases were observed, an excess of 6%.

Prostate cancer is now the most frequently diagnosed cancer in men in the UK. Worldwide, prostate cancer has been diagnosed with increasing frequency over the recent decades (Coleman *et al*, 1993). The extent to which the observed increase represents an increase in the inception of the disease as opposed to an artefact of increased detection remains debatable.

In the UK, prostate-specific antigen (PSA) testing became generally available in 1991 but has not been recommended for mass screening. *Ad hoc* case finding by PSA testing, and increased PSA testing of men who present with lower urinary tract symptoms (LUTS), both have the capacity to inflate registration rates for prostate cancer. In men, the prevalence of urological symptoms increases with age and the increased availability of PSA testing may be leading to diagnosis of prostate cancer which either would not have arisen clinically or would not have come to clinical attention later in life.

This study of the incidence of prostate cancer in the three counties of Norfolk, Suffolk and Cambridgeshire (NSC) between 1971 and 2000, sought to assess the effect of PSA testing on prostate cancer registrations during the 1990s.

## MATERIALS AND METHODS

Extracts from the records of all men resident in NSC with prostate cancer diagnosed from 1/1/1971 to 31/12/2000 were obtained from the Eastern Cancer Registry and Information Centre (ECRIC). Mid-year population estimates for the three counties for each year from 1971 to 2000, stratified by sex and age were obtained from the Office of National Statistics (ONS).

Age-specific and age-standardised incidence rates (using direct standardisation and the European Standard Population) were calculated for each 5-year period of observation beginning with 1971–1975. This was followed by age-period-cohort analysis, with ten 5-year age groups (40–44 to 85–89 years), six 5-year periods of observation (1971–1975 to 1996–2000) and 15 approximate birth cohorts, represented by central years of birth from 1886 to 1956.

Poisson regression of the incidence on age, period and cohort was performed. To estimate the separate effects of age (A), calendar period (P) and birth cohort (C) on the incidence trend, a series of models were fitted sequentially, using the methods described by Clayton and Schifflers (1987a, 1987b). The first model represented the null hypothesis of an age effect with no temporal variation, while the second model included effects of age and a regular drift, a smooth trend in incidence which could be attributable to either period or cohort influences. Subsequently, age-period (AP), age-cohort (AC) and age-period-cohort (APC) models were fitted. Models were compared using deviance *χ*^2^ tests. Although the parameters from the APC model cannot be interpreted due to unidentifiability, the related significance test remains appropriate.

The analysis was repeated restricted to 1971 to 1990, before PSA testing was introduced. The best fitting model was used to predict incidence from 1991 to 2000. The number of predicted cancer cases by age group was calculated and compared to the observed. Analysis was performed using STATA 7.0.

## RESULTS

Between 1971 and 2000, 22 418 men of mean age (s.d.) 74 (8. 5) years with newly diagnosed prostate cancer were registered in NSC. Table 1 shows the incidence rates of registered prostate cancer in NSC by age and calendar period. The corresponding numbers of cases and person-years are available from the authors. The approximate birth cohorts can be identified as diagonals of the table, descending from left to right. The increase over time was most marked for ages 50–69. The variable increase in the incidence rates over time by age suggests that there may be cohort effects.

The age-standardised rates increased from 32/100 000 men-years in 1971 to 89/100 000 men-years in 2000, an almost threefold increase. In 1991, the age-standardised incidence rate was 57/100 000 men-years. Thus, a similar change was observed in the last 10 years to that observed for the first 20 years.

Table 2 shows the relative risks (RR) for period adjusted for age and cohort adjusted for age (AP and AC models). Compared to the period 1971–1975, the age-adjusted relative risk of prostate cancer registration increased to 2.30 (95% CI 2.17–2.43) for the period 1996–2000. Compared to the 1886 birth cohort, the RR increased to 14.16 (95% 3.55–56.55) for the 1956 birth cohort. From the table, one can see an approximate 15% increase in incidence per successive cohort up to birth year 1921, and a 30–40% increase per successive cohort from birth years 1926 to 1946. The more recent periods and/or the younger birth cohorts had increased incidence of prostate cancer.

The ‘drift’ parameter of Clayton and Schifflers (1987a), a trend in incidence which could be interpreted as either a period or cohort effect, was highly significant, a 19% increase in incidence per 5-year period or birth cohort (RR=1.19, 95%CI 1.17–1.20). The effect of cohort adjusted for age and drift was strongly statistically significant (*P*<0.001). The period effect was not significant after adjustment for age and drift (*P*=0.1). The age-period-cohort analyses of prostate cancer thus indicated a stronger dependency on cohort than on period, but the full APC model provided the best fit to the data.

Restricting the analysis to the period 1971–1990, the effects of period adjusted for age and drift (*P*>0.1) and cohort adjusted for age and drift (*P*>0.1) were not significant. This indicated that a single trend of increasing risk (with time or cohort) was sufficient to model changes in incidence. The drift parameter in the 1971–1990 data indicated a 16% increase in incidence per 5-year period or birth cohort (RR=1.16, 95% CI 1.14–1.19).

**Figure 1:**
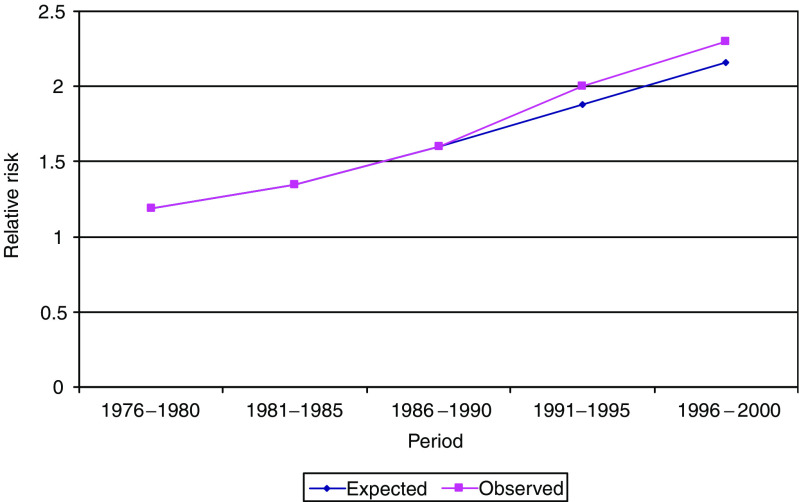
Expected risk of prostate cancer 1976–2000, relative to rates in 1971–1975, estimated using pre-1990 data, compared with the observed relative risk after 1990.

To assess whether the advent of PSA testing in 1991 was accompanied by a change in the incidence trend, we used the 16% drift parameter from the data up to 1990 to predict incidence from 1991 to 2000. This was then compared with the observed incidence. Observed and predicted numbers are shown in Table 3. The prediction underestimated the 1991–2000 incidence by around 6% (*P*<0.001) (Figure 1).

## DISCUSSION

This study shows that the incidence of prostate cancer in NSC has been increasing from 1971 and that this increase has been accelerating in recent years. Incidence trends of 1971–2000 show cohort effects. The number of cases since 1991 exceeds by 6% the number predicted from pre-1991 trends. The fact that actual incidence was 6% higher than predicted suggests that changes in risk or in diagnostic practice took place specifically in the 1990's leading to a larger number of diagnosed cases (585 extra cases).

The observed increases in incidence in NSC are similar to the trends in England and Wales (Majeed *et al*, 2000; Quinn *et al*, 2001). Increases in the incidence before the introduction of PSA testing have been reported in other European countries (Gronberg *et al*, 1994; Harvei *et al*, 1996; Moller, 2001) and the USA (Potosky *et al*, 1990).

Age-period-cohort modelling using all the data from 1971 to 2000 indicated that cohort effects were taking place, that is, changes over time were not happening uniformly across the age range. Increasing incidence was particularly pronounced for the birth cohorts from 1926 to 1946 and for the 50–69 age groups in the 1990s.

To our knowledge, there are no previous reports of APC modelling of prostate cancer incidence in the UK. A study from Denmark (Moller, 2001) from 1943 to 1996 indicated a stronger dependency on period than on birth cohort. An APC analysis from five French administrative areas, covered by population-based registry, from 1982 to 1995 showed period effects and a small but significant cohort effect (Chirpaz *et al*, 2002).

If the difference between observed and predicted number of cases was due to a new detection modality, such as PSA testing, then we might expect the effect of this detection modality to be larger than 6% especially after 1995. The apparent gap remained 6% for both periods of 1991–1995 and 1996–2000. This may be because the increase in PSA testing was compensated for by a reduction in detection from transurethral resection of the prostate (TURP). Age-specific patterns in the increased use of PSA and decreased use of TURP may also be responsible for the greater increase in incidence in those aged under 70 years. Our companion paper with local data on individual exposure to PSA testing also addresses the age issue (Pashayan *et al*, 2006).

It should be noted that while the excess in 1991–2000 is significant and almost certainly a real phenomenon, and probably largely due to increased availability of PSA testing, it is modest. Further quantification of excess incidence due to PSA testing is needed.

## Figures and Tables

**Table 1:** Incidence of registered prostate cancer per 100 000 men-years by age group and period of diagnosis, NSC, 1971–2000

	**Period of diagnosis**					
**Age group**	**1971–1975**	**1976–1980**	**1981–1985**	**1986–1990**	**1991–1995**	**1996–2000**
40–44	1	0	0	0	1	1
45–49	2	1	2	3	2	3
50–54	8	6	8	8	14	23
55–59	23	29	34	35	46	76
60–64	48	75	89	92	119	177
65–69	120	151	171	189	267	371
70–74	236	255	323	365	432	525
75–79	375	437	469	548	692	734
80–84	445	527	566	807	934	847
85–89	629	735	707	897	1088	1004
						
Crude rate	83	106	128	153	191	223

**Table 2:** Relative risks (RR) and 95% confidence intervals (CI) for the period and cohort effects adjusted for age, NSC, 1971–2000

	**RR**	**95% CI**	
*Period*			
1971–1975	Reference	—	—
1976–1980	1.19	1.11	1.27
1981–1985	1.35	1.27	1.45
1986–1990	1.60	1.51	1.71
1991–1995	2.00	1.89	2.12
1996–2000	2.30	2.17	2.43
			
Cohort (central birth year)			
1886	Reference	—	—
1891	1.03	0.83	1.28
1896	1.16	0.95	1.42
1901	1.34	1.10	1.63
1906	1.58	1.30	1.92
1911	1.80	1.49	2.18
1916	2.06	1.70	2.50
1921	2.42	1.99	2.94
1926	3.06	2.51	3.73
1931	4.16	3.40	5.09
1936	5.25	4.24	6.51
1941	7.58	5.94	9.69
1946	10.47	7.53	14.55
1951	8.72	4.35	17.50
1956	14.16	3.55	56.55
			
‘Drift’, RR per 5 years	1.19	1.17	1.20

**Table 3:** Observed and expected number of cases of prostate cancer in NSC, periods 1991–1995 and 1996–2000 by age group, based on trends for the period 1971–1990

	**Observed**	**Expected**	**SE (expected)**	**Observed – expected**
1991–1995				
40–44	2	2	0.45	0
45–49	7	11	0.21	−4
50–54	42	31	0.12	11
55–59	122	117	0.06	5
60–64	305	279	0.04	26
65–69	650	553	0.03	97
70–74	937	919	0.03	18
75–79	1003	946	0.03	57
80–84	881	805	0.03	76
85–89	414	403	0.04	11
Sub total	4363	4067		296
				
1996–2000				
40–44	3	2	0.448	1
45–49	10	13	0.211	−3
50–54	87	46	0.118	41
55–59	224	150	0.066	74
60–64	474	341	0.049	133
65–69	922	656	0.041	266
70–74	1146	1076	0.038	70
75–79	1264	1308	0.037	−44
80–84	819	960	0.039	−141
85–89	476	584	0.049	−108
Sub total	5425	5136		289
				
Total	9788	9203		585
